# Rapid or Slow Time to Brain Death? Impact on Kidney Graft Injuries in an Allotransplantation Porcine Model

**DOI:** 10.3390/ijms20153671

**Published:** 2019-07-26

**Authors:** Thomas Kerforne, Sébastien Giraud, Jérôme Danion, Raphael Thuillier, Pierre Couturier, William Hebrard, Olivier Mimoz, Thierry Hauet

**Affiliations:** 1 CHU Poitiers, Coordination des prélèvements d’organe et de tissus, F-86000 Poitiers, France; 2 CHU Poitiers, Département d’anesthésie-réanimation, F-86000 Poitiers, France; 3 INSERM U1082 (IRTOMIT), F-86000 Poitiers, France; 4 CHU Poitiers, Service de Biochimie, F-86000 Poitiers, France; 5 Faculté de Médecine et Pharmacie, Université de Poitiers, F-86000 Poitiers, France; 6 CHU de Poitiers, Service de Chirurgie viscérale, F-86000 Poitiers, France; 7 IBiSA ‘plate-forme MOdélisation Préclinique - Innovations Chirurgicale et Technologique (MOPICT)’, Domaine Expérimental du Magneraud, F-17700 Surgères, France; 8 INRA, Unité expérimentale Génétique, expérimentations et systèmes innovants (GENESI), Domaine Expérimental du Magneraud, F-17700 Surgères, France; 9 CHU Poitiers, Service d’accueil des urgences, SAMU-SMUR, F-86000 Poitiers, France

**Keywords:** brain death, kidney, transplantation, oxidative stress, allostasis, nuclear factor erythroid-2-related factor 2, mechanistic target of rapamycin

## Abstract

The use of donors deceased after brain death (DBD) with extended criteria in response to the shortage of grafts leads to the removal of more fragile kidneys. These grafts are at greater risk of not being grafted or delayed function. A better knowledge of the pathophysiology of DBDs would improve this situation. There is a difference between the results from animal models of DBD and the clinical data potentially explained by the kinetics of brain death induction. We compared the effect of the induction rate of brain death on the recovery of post-transplant renal function in a pig model of DBD followed by allografts in nephrectomized pigs. Resumption of early function post-transplant was better in the rapidly generated brain death group (RgBD) and graft fibrosis at three months less important. Two groups had identical oxidative stress intensity but a greater response to this oxidative stress by SIRT1, PGC1-α and NRF2 in the RgBD group. Modulation of mechanistic target of rapamycin (mTOR) stimulation by NRF2 would also regulate the survival/apoptosis balance of renal cells. For the first time we have shown that an allostatic response to oxidative stress can explain the impact of the rapidity of brain death induction on the quality of kidney transplants.

## 1. Introduction

At this time, renal transplantation is the recommended treatment for end-stage kidney disease. Unfortunately, there is a persistent lack of kidney grafts, with a mismatch between the number of new patients registered on a transplant waiting list and the number of grafts available. Even though over recent years, several strategies have been drawn up to alleviate this gap with new types of organ donations from marginal donors and controlled and non-controlled donors having died after circulatory collapse, brain death donors (DBD) remain the principal source of grafts in the world [1,2].

However, DBD organs are exposed to injuries induced by brain death (BD) increasing their sensitivity to ischemia-reperfusion (IR) injury and compromising organ quality. Indeed, these injuries contribute to primary non-function and delayed graft function. This phenomenon is aggravated by the growing number of “expanded criteria” donors [3]. In this context, better understanding and improvement of DBD management protocols prior to organ explantation is of utmost importance in view of upgrading organ quality. Comprehension of the different phenomena secondary to brain death occurrence and their repercussions on potential grafts is essential to the elaboration of management protocols increasing the quantity and improving the quality the donor organs susceptible to being extracted.

In the literature the usual animal brain death models are essentially mouse models characterized by brutally induced brain death, and they present limits, particularly as regards to the kidneys [4]. At times, the data issued from these models are contradictory to findings in health clinics for humans [5].

A recent experimental model was focused on the impact of the kinetics of brain death on the different intra-abdominal organs (kidney and liver), and the results highlighted appreciable difference [6]. However, this study was based on a mouse model, and its results are to be interpreted with caution, especially insofar as the grafts from the donors were finally not transplanted.

Wishing to provide clinicians with potential intervention routes in brain death donor (DBD) management, we reproduced near clinical brain death (BD) conditions in a preclinical porcine model of kidney BD donors prior to transplantation. In fact, due to anatomy and physiology similar to humans, the pig is of pronounced interest as a preclinical model [4].

Our objective was to determine the time-dependent effects of rapid brain death induction (RgBD) as opposed to slow brain death induction (SgBD) on the kidney during 4h donor management after BD declaration. We opted for four hours of management subsequent to brain death based on the results reported by Schuurs et al. [7], who showed that after four hours, considerable expression of the genes coding for the pro and anti-inflammatory responses secondary to brain death was to be observed. 

**Figure 1:**
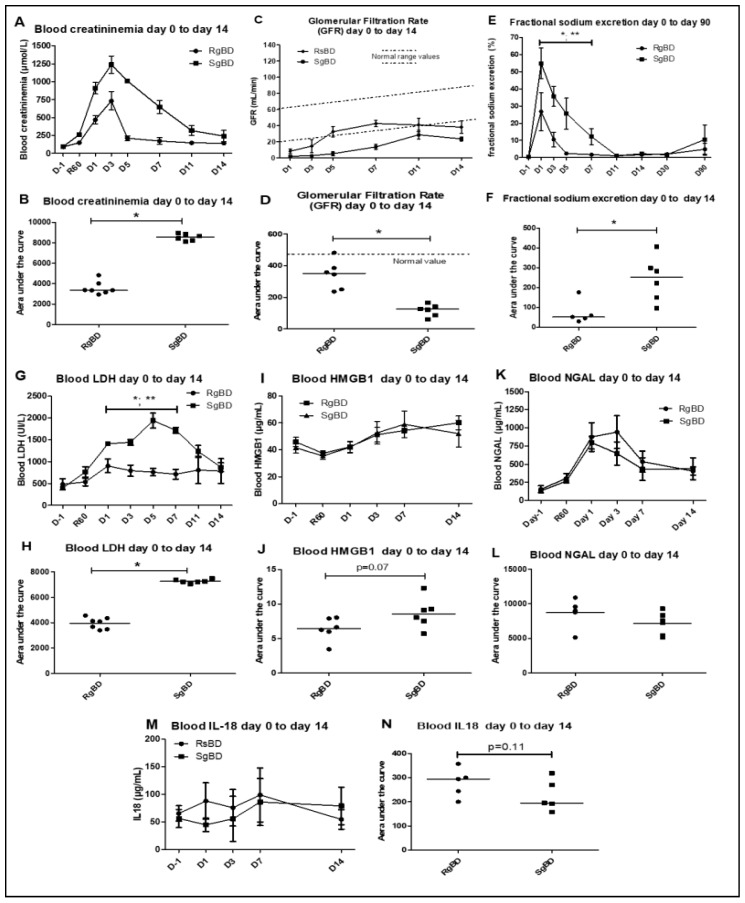
Renal function evaluation from day 0 to day 14 after transplantation. Renal function evaluation of the first 14 days after allotransplantation of kidneys subjected to rapid brain death induction (RgBD) or slow brain death induction (SgBD). Blood creatinine (**A**,**B**), glomerular filtration rate in mL/min (**C**,**D**), fractional sodium excretion (**E**,**F**), blood LDH (**G**,**H**), blood high mobility group box 1 (HMGB1; **I**,**J**), blood NGAL (**K**,**L**) and blood IL-18 (**M**,**N**) were analyzed from the day before transplantation (**D**–**L**) to day 14 after transplantation. (*n* = 5 to 7 per group). Kinetic results (**A**,**C**,**E**,**G**,**I**,**K**,**M**) are expressed as mean ± SD, statistical analysis was performed with Kruskal–Wallis multiple-comparison Dunn’s test. Areas under the curve (AUC; **B**,**D**,**F**,**H**,**J**,**L**,**N**) are expressed in a scatter plot with the median, statistical analysis was performed with a Mann–Whitney test. * RgBD *vs* SgBD; ** *vs* D-1 *p* < 0.05.

We consequently explored the impact of RgBD and SgBD on critical pathways determining kidney graft quality, specifically targeting oxidative stress [8] and post-transplant outcome (Figure 1).

We highlighted a difference in early function recovery and observed more severe chronic injuries in pigs grafted with kidneys from slow generated brain death (SGBD). This observation validates previous findings in mouse models and should lead to proposal of animal models better suited to the models being tested.

## 2. Results

### 2.1. Graft Function Recovery

#### 2.1.1. Early Function Recovery from D0 to D14; Markers of Endothelial, Renal Tubular Lesions and Tissue Injuries

We observed no difference in diuresis recovery between the two groups. However, the pigs grafted with group RgBD kidneys presented better early function recovery, with post-graft creatinemia from D1 and D5 significantly lower than that of the pigs grafted with SgBD kidneys (Figure 1A). At post-transplantation D3, the SgBD group presented a significantly higher creatinemia peak (*p* = 0.0012) than the RgBD group (1239 μmol/L vs. 733 μmol/L respectively). Area under the curve (AUC) creatinine kinetics from D0 to D14 was significantly greater in the SgBD group than in the RgBD group (Figure 1B). The glomerular filtration rates (GFR) were significantly different between the two groups, with a better filtration rate in the RgBD group from D1 to D14 (Figure 1C,D). Fractional excretion of sodium was significantly higher in the RgBD group than in the SgBD group from D1 to D5 (Figure 1E,F).

In terms of markers of cell necrosis, concentrations of blood LDH and blood HMGB1 (high mobility group box 1) appeared higher (AUC analyses) in the SgBD group from D1 to D14 (Figure 1G–J). On the contrary, no significant difference in blood neutrophil gelatinase-associated lipocalin (NGAL) level between the two groups was observed during the first 14 days of transplantation (Figure 1L). Concentrations of blood NGAL (neutrophil gelatinase-associated lipocalin) increased significantly from 60 min to D1 after renal unclamping in the RgBD group and peaked at D2 in the SgBD group (Figure 1K). In the two groups, decrease levels, although without normalization, were observed at D14. Concentrations of blood IL-18 did not increase significantly in the two groups between unclamping and D14 (Figure 1M,N). 

#### 2.1.2. Late Function Recovery (J90), Injury Markers and Fibrosis

**Figure 2:**
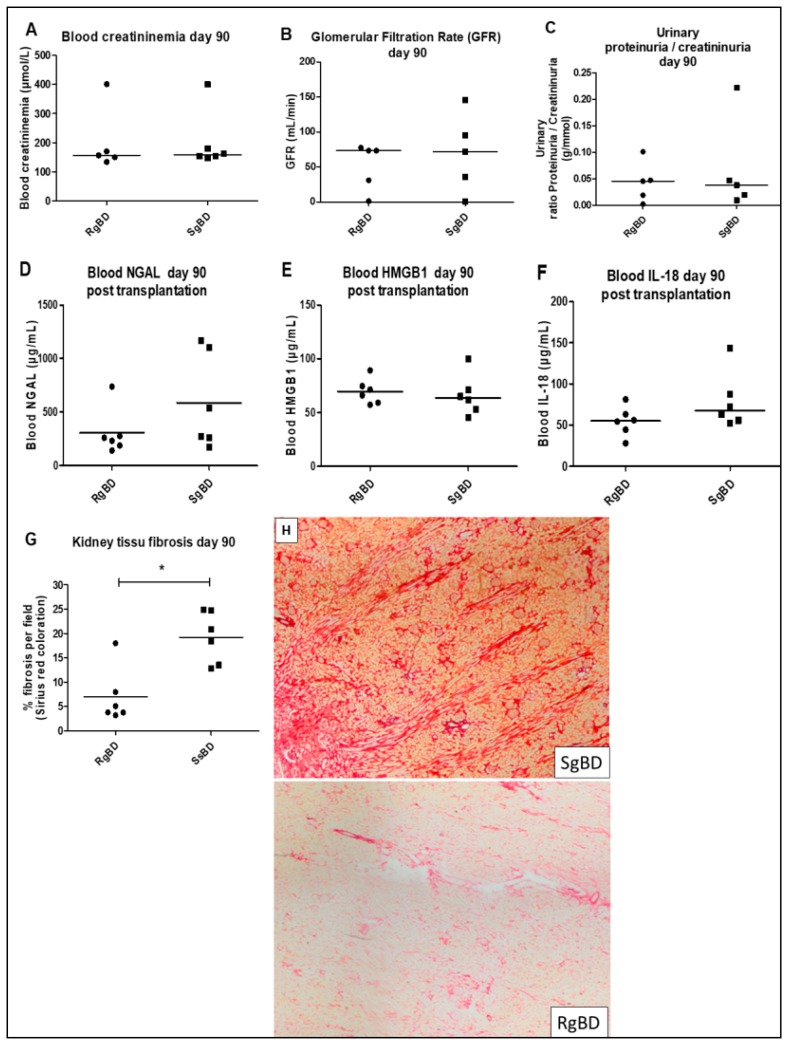
Renal function evaluation at day 90 after transplantation. Renal function evaluation at day 90 after allotransplantation of kidneys submitted to rapid brain death induction (RgBD) or slow brain death induction (SgBD). Blood creatinine (**A**), glomerular filtration rate in mL/min (**B**), ratio proteinuria/creatininuria (**C**), blood NGAL (**D**), blood HMGB1 (**E**), blood IL-18 (**F**) and kidney tissue fibrosis (**G**) evaluated by histological red Sirius staining magnification × 6.3 (**H**) analyzed at day 90 after transplantation. (*n* = 6 per group). Results are expressed in a scatter plot with the median, statistical analysis was performed with the Mann–Whitney test. * *p* < 0.05.

At D90 after transplantation we observed no significant difference between groups RgBD and SgBD concerning blood creatinine level, glomerular filtration rates or proteinuria/creatininuria ratio (Figure 2A–C). Furthermore, blood HMGB1, NGAL and IL-18 concentrations did not significantly differ between the two groups (Figure 2D–F). However, there was significantly more fibrosis in the kidneys of the SgBD group than in the RgBD group (Figure 2G,H).

### 2.2. Donor Management

#### 2.2.1. Donor Management: Hemodynamic Stability, Oxygen Transport and Biological Stability

After induced brain death, all donor animals presented a Cushing reflex with maximal cardiac output and arterial hypertension, which was followed by a fall in arterial pressure and arterial flow. The animals were stabilized by the introduction of norepinephrine and vascular filling maintaining mean arterial pressure at 65 mmHg and cardiac index between 2 and 3 L/min. Hourly diaresis by reanimation did not significantly differ between the two groups (Table 1). Lactate significantly increased in the two groups after 60 min of reanimation and subsequently diminished in both of them (Table 1). 

**Figure 3:**
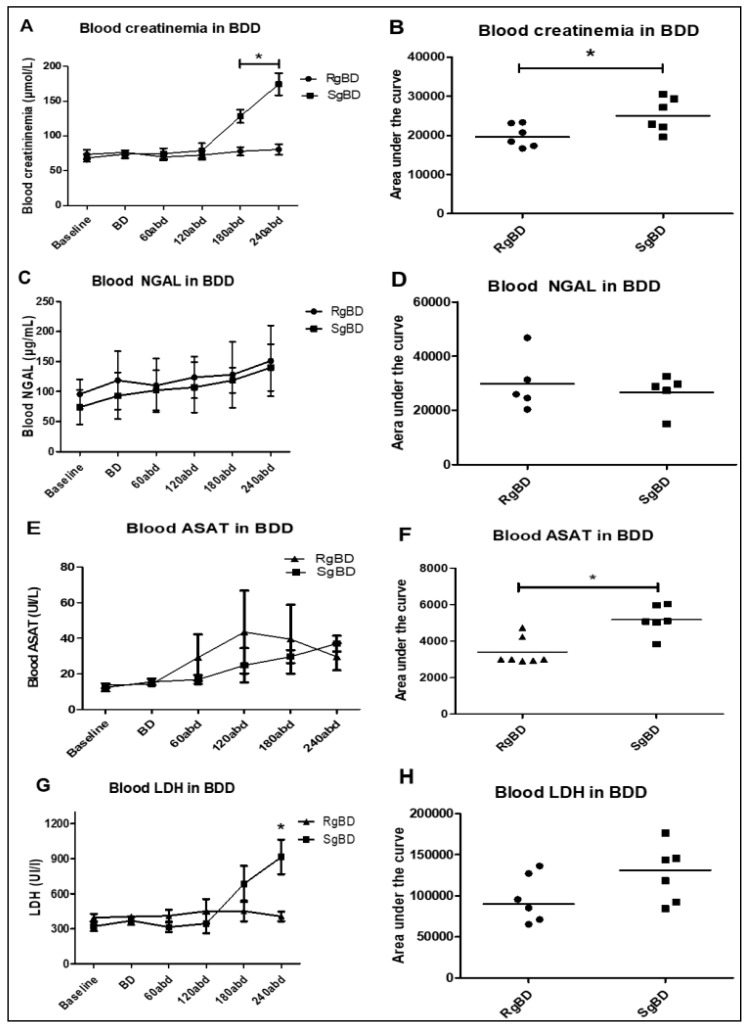
Donor management: Biological stability of the two groups during reanimation. Evaluation of kidney injury marker evolution during BD donor management after rapid brain death induction (RgBD) or slow brain death induction (SgBD). Blood creatinine (**A**,**B**), blood NGAL (**C**,**D**), blood aspartate aminotransferase activity (ASAT) (**E**,**F**) and blood LDH (**G**,**H**) concentration blood evolution, investigated at control time (baseline), brain death time (**B,D**), and per donor management at 60, 120, 180 and 240 min after brain death (abd; *n* = 5–6 per group). Kinetic results (**A**,**C**,**E**,**G**) are expressed as mean ± SD, statistical analysis was performed with a Kruskal–Wallis multiple-comparison Dunn’s test. Areas under the curve (**B**,**D**,**F**,**H**) are expressed in a scatter plot with the median, statistical analysis was performed with a Mann–Whitney test. * *p* < 0.05.

From a biological standpoint, a significant increase in creatinemia following three hours of reanimation was observed in the SgBD group, as was an increase in blood LDH (Figure 3A–D). The circulating level of NGAL increased non-significantly during the 4 h of reanimation, and there was no significant difference between the animals of the RgBD and the SgBD groups (Figure 3E,F). Aspartate aminotransferase activity (ASAT) blood levels increased non-significantly during the two hours of reanimation, and then decreased in the RgBD group while continuing to increase in the SgBD group. In comparison of the areas under curve (AUC) of the kinetic parameters of ASAT between the two groups, AUC for the SgBD group was significantly higher than for the RgBD group (Figure 3G,H). 

#### 2.2.2. Donor Management: Markers of Oxidative Stress and Antioxidant Response

**Figure 4:**
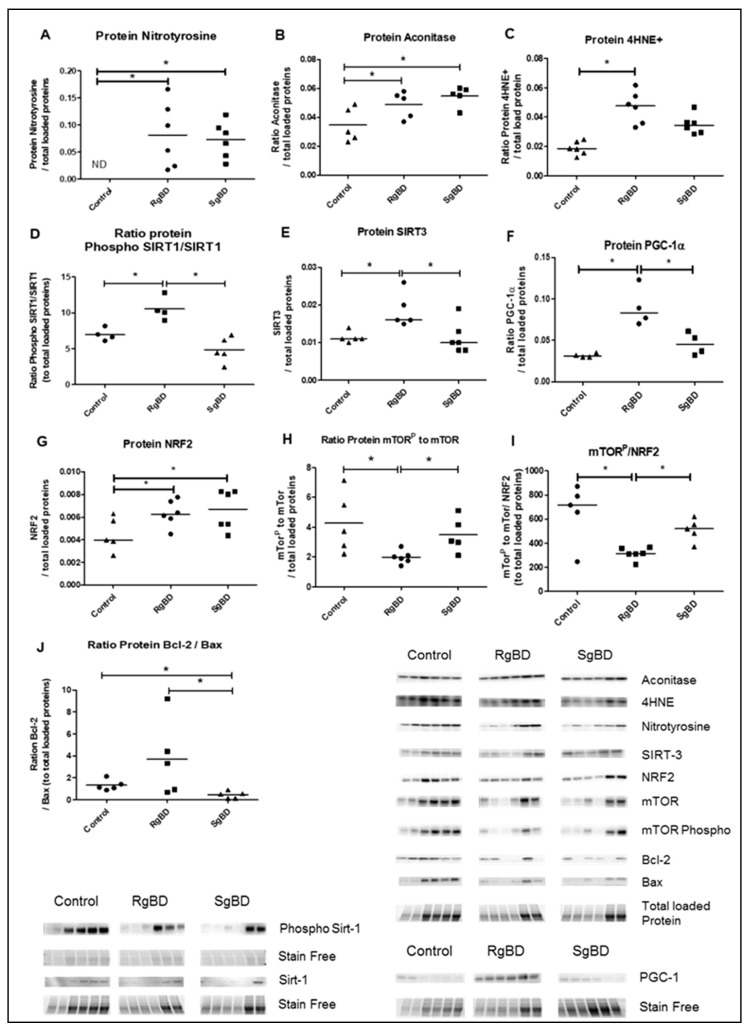
Donor management: Oxidative stress and response to oxidative stress by SIRTs-PGC-1a-NRF2-mTOR path and mitochondrial integrity at the end of reanimation. Protein expression level of nitrotyrosine (**A**), aconitase (**B**), 4HNE (**C**), ratio phosphorylated SIRT-1 to total SIRT-1 (**D**), SIRT-3 (**E**), PGC-1a (**F**), NRF2 (**G**), ratio phosphorylated mTOR to total mTOR (**H**), ratio phosphorylated mTOR to NRF2 (**I**) and ratio Bcl2 to Bax (**J**), evaluated by western blot at the end of 4 h donor management in renal tissue of rapid brain death induction (RgBD) and slow brain death induction (SgBD) groups versus control kidney (*n* = 5–6 per group). Results are expressed in a scatter plot with the median, statistical analysis was performed with a Kruskal–Wallis multiple-comparison Dunn’s test. * *p* < 0.05.

At the conclusion of reanimation, protein expression in renal tissue marked the intensity of oxidative stress. Nitrotyrosine, aconitase and 4HNE were overexpressed in the two groups as compared to the uncontrolled kidneys without any significant difference between the SgBD and the RgBD groups (Figure 4A–C). The proteins implicated in response to oxidative stress, ratio of phosphorylated SIRT-1 to total SIRT-1, SIRT-3 and PGC-1a, were significantly more expressed in the RgBD group than in the SgBD group (Figure 4D–F). The antioxidant protein NRF2 (also called NRLF2) was overexpressed in the two groups at the conclusion of reanimation without any significant difference between the SgBD and the RgBD groups (Figure 4G). The ratio of phosphorylated mechanistic target of rapamycin (mTOR) to total mTOR was significantly lower in the RgBD groups than in the control and the SgBD groups (Figure 4H). The phosphorylated mTOR/NRF2 ratio was significantly lower in the RgBD group than in the SgBD group (Figure 4I). After 4 h of reanimation, the Bcl2/Bax protein ratio, a marker of anti-apoptotic mitochondrial protein balance, was significantly higher in the RgBD group than in the SgBD group (Figure 4J). 

#### 2.2.3. Donor Management: Markers of Endothelial Activation

**Figure 5:**
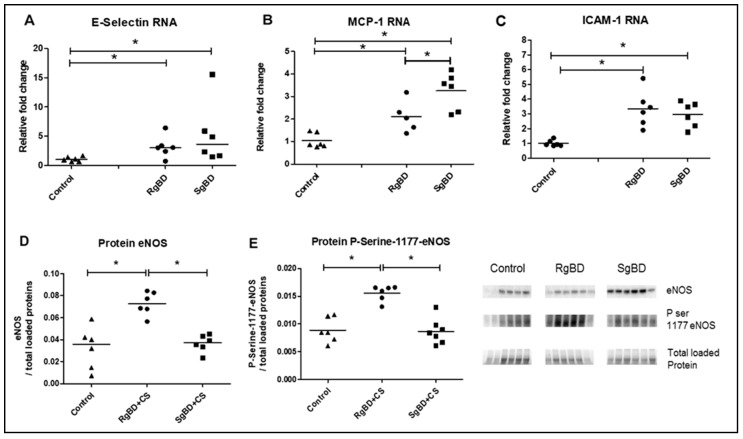
Donor management: Endothelial activation; genomic expression of adherence molecules and protein expression of endothelial nitric oxide synthase (eNOS) and P-Serine-1177-eNOS at end of reanimation. RNA expression level of E-Selectin (**A**), CCL2/MCP-1 (**B**) and ICAM-1 (**C**), and protein expression level, by western blot, of eNOS (**D**) and ratio Serine 1177 phosphorylated eNOS to eNOS total (**E**) evaluated in renal tissue of rapid brain death induction (RgBD) and slow brain death induction (SgBD) groups at the end of cold storage following 4 h donor management (called RgBD + CS and SgBD + CS) versus control kidney (*n* = 5–6 per group). Results are expressed in a scatter plot with the median, statistical analysis was performed with a Kruskal–Wallis multiple-comparison Dunn’s test. * *p* < 0.05.

Messenger RNA coding for adhesion protein E-selectin, and CCL2/MCP-1 monocyte attraction were significantly overexpressed in the two BD groups after 4 h of reanimation. Further, CCL2/MCP-1 mRNA expression was increased to a significantly greater extent in the SgBD group (Figure 5A,B). The mRNA of the high-adhesion molecule ICAM-1 was significantly more expressed in the two BD groups after 4 h of reanimation than in the control kidneys (Figure 5C). Regarding protein expression, at the end of the static conservation period, the endothelial nitric oxide synthase (eNOS) and the serine 1177 phosphorylated eNOS vasoreactivity proteins were significantly more expressed in the RgBD group than in the SgBD and the control groups (Figure 5D,E).

## 3. Discussion

At present we are lacking in fundamental data that would improve the treatment of brain death donors (DBD) and achieve early characterization of future kidney grafts during the phase of donor reanimation. The relevant scientific literature presents contradictory clinical and pre-clinical data featuring sizeable differences between the data obtained in humans and those in animal models. The difference may be explained by a limited choice of usable animal models, namely mouse models with rapid, even instantaneous systemic inflammation during BD [5]. While mouse models in renal transplantation are imperfect, pig models are anatomically and physiologically more satisfactory [4]. Moreover, one of the other limitations in pre-clinical studies in rodents consists in systemic inflammatory response during BD, which does not correspond to usual clinical situations in which several hours elapse between a patient’s brain injury and occurrence of BD. That being said, a recent study using a mouse model compared the impact of different speeds of BD induction. While the results are of interest, showing a detrimental effect of slow-speed induction of brain death on the biological parameters of renal injuries [6], this study did not purport to evaluate post-transplant function recovery in kidney grafts. The authors show an increased oxidative stress and apoptosis in the organs of the “slow” BD induction group as compared to the “rapid” induction group. As regards plasma creatinine concentration, once again it was higher during reanimation in the slow induction than in the rapid induction animal groups. The one hemodynamic parameter considered in this study was mean arterial pressure (MAP), and its mean values did not significantly differ between the two groups.

Here, in our preclinical study of a BD pig model, we recorded comparable results, with a deleterious impact of slowly induced BD on post-transplant function recovery in kidney grafts. The originality of our work consists in (a) the use of a BD pig model, (b) the transplantation of kidneys from said models, and (c) follow-up lasting up to three months, a time span enabling us to complement data on recovery of initial kidney function with data on chronic injuries. 

Our results show that primary graft function recovery was pronouncedly less satisfactory in pigs grafted with kidneys originating in the “slow” group than with pigs grafted with kidneys originating in the “rapid” group, who displayed markedly superior post-transplantation plasma creatinine levels during the first week. This difference is also found in post-transplantation kinetics up until D14. We also investigated markers of inflammation, such as IL-18, which did not demonstrate a difference between the groups; further we attempted to measure TNFα, IL-6 and IL-10, however these were not detected. This may be due either to dilution of the cytokine in the systemic blood flow or possibly to the incompatibility of the kit with the pig, a problem that is unfortunately common in this species.

In human clinical cases, the DBD parameters liable to impact primary functional recovery are: Preexisting pathological states along with age, cardiovascular comorbidity factors (expanded criteria donors) [3], donor hemodynamic instability [9,10,11,12] and cold ischemia duration. In the controlled setting of our pre-clinical study, all of the pigs had the same age (14 weeks) and were devoid of any pathological state prior to experimentation; moreover, reanimation duration was uniform, and the different grafts underwent cold ischemia in static conservation for the same length of time. Only the BD hemodynamics was more unstable in the RgBD group, and the variations were statistically identical.

The quality of post-BD hemodynamic reanimation was identical in the two groups of animals, and the results were similar regarding the determinants of oxygen transport (cardiac output, oxygen saturation of arterial hemoglobin and partial pressure of oxygen). Moreover, the arterially extracted blood lactate in the two groups observed the same increase kinetics at the moment of brain death and after 60 min of reanimation, a result underlining a balance between the supplies and needs of oxygen in the two groups. A possible difference in oxygen transport during reanimation consequently does not explain the observed differences between the groups regarding function recovery. On the other hand, we observed a trend towards increased LDH plasma concentration in the “progressive” group; it is explained by the peculiarities of kinetics during brain death, all other factors remaining experimentally identical. This cell lesion marker was associated with a significant ASAT increase, which may function as a marker of renal tubular lesion [13] and be considered as deterioration of the renal function in association, notwithstanding satisfactory hemodynamics, with plasma creatinine increase. When it had been decided that reanimation would go on for four hours, the decision was based on the fact that in 100% of procedures, this time lapse allowed for stabilization of a given hemodynamic state; moreover, it allowed for presentation of maximum tissular expression of inflammatory markers [7].

We explored oxidative stress and response to oxidative stress for two fundamental reasons: (a) Oxidative stress is associated with the occurrence and aggravation of numerous chronic renal illnesses [14]; data collected in works by Stiegler et al. highlighted activation of oxidative stress subsequent to BD occurrence [8]. In their study, renal nitrotyrosine through immunohistochemistry testing was shown to increase after BD. Moreover, oxidative stress in a donor tends to delay function recovery [15].

In the study by Stiegler et al., a number of markers, parameters of response to oxidative stress (superoxide dismutase 2, gluthation synthetase, peroxisome proliferator-activated receptor alpha, oxidative stress responsive 1 and gluthation peroxidase 3) were analyzed, but only as regards to their mRNA. The authors found no increased expressions of these genes in the kidneys of DBDs; this is probably due to the fact that response to oxidative stress in the short time span of reanimation is mainly induced by the activation of anti-oxidizing proteins. In our study, we focused on three markers reflecting the intensity of oxidative stress at the level of renal tissue: 4HNE, aconitase and nitrotyrosine. In order to evaluate response to oxidative stress, we analyzed the expression of anti-oxidizing proteins by Western blot (Sirtuins, PGC1α and NRF2), which is more informative than mRNA.

Our results have shown that the kinetics of BD induction exerted little or no influence on the intensity of oxidative stress; levels of 4HNE, nitrotyrosine and aconitase were similarly overexpressed in the two BD groups. However, the kinetics of BD induction seemed to influence the response to oxidative stress mediated by Sirtuin 1 and 3 and PG1alpha. However, NRF2 was similarly overexpressed in the two groups. In addition, we studied the degree of phosphorylated mTOR expression in total mTOR. mTOR is a major, indispensable actor in numerous cellular mechanisms, particularly as regards energy regulation in renal tubular cells and fibrosis generation secondary to an aggression inducing delayed graft function loss [16]. In the explanted kidneys of the RgBD group, activation of the mTOR protein was significantly reduced. While mTOR modulation by the ROS produced during oxidative stress is confirmed by the relevant scientific data, it can take on the form of either upregulation via AMPK [17] or of downregulation via FoxO3 [18]. On this topic, recent publications have highlighted direct regulation of mTOR by NRF2 [19] as well as double downregulation involving mTOR and NRF2 [20]. mTOR detection is also concordant with our apoptosis results [21], which demonstrated higher apoptosis in the RgBD.

Our hypothesis was that the observed differences in primary function recovery in kidney grafts according to BD kinetics was associated not with a difference in oxidative stress intensity, but rather with modulation of anti-oxidative response intensity leading to modulation of mTOR by NRF2. This difference in secondary adaptation to an aggression corresponds to the phenomenon of allostasis, i.e., adaptation of a system to new environmental conditions. To our knowledge, this is the first time that a donor allostasis mechanism has been described. The observed difference in allostatic adaptation could originate in the protective phenomena initiated by shear stress, insofar as it has been demonstrated that sheer stress has a protective effect, particularly through eNOS activation [21], and that the effect may entail the intervention of SIRT-1 [22,23]. Part of the originality of this paper consists in our having highlighted more pronounced expression of the ratio of phosphorylated eNOS to total eNOS in the rapidly generated brain death (RgBD) group. We may thus hypothesize that post-BD, the renal cells were subject to intensified shear stress involving SIRT-1 activation, which might help to explain our results. These organ level modifications of vascular homeostasis did not have observable impact on the post-BD hemodynamic parameters, possibly because such local change is unlikely to have measurable effect at the systemic level, especially measured by the typical tools of hemodynamics monitoring, which lack precision. Finally, the hemodynamic phenomena secondary to BD were more condensed in the RgBD group (5 min) than in the in the “slow” SgBD group (90 min), which strengthens the hypothesis of an involvement of shear stress.

Observation of eNOS phosphorylation and modulation of vascular homeostasis, which is of better quality in the RgBD group, is in line with the observed difference in fibrosis shown herein: As demonstrated in earlier studies from our lab [24,25], ischemia reperfusion targets mainly micro vessels, with deleterious consequences on the long term, including fibrosis development.

The potential interest of this work consists in its possibly explaining, for reasons other than the imperfect use of mouse models, the divergencies of the results reported in the literature. Indeed, analysis has often been based on evaluation of intensity of aggression rather than intensity of the response to the aggression of the regulation/response systems.

This work presents some limits, particularly as regards extrapolation of the results to humans and to the kidneys of donors presenting preexisting lesions, given the fact that mTOR is regulated in different ways in different renal pathologies [16]. Even if our results possess some external validity insofar as they confirm those of Schuur et al., other experimentations remain necessary.

Were our results to be confirmed, several possible applications could be envisioned, particularly therapeutic utilization of the mTOR and/or NRF2 modulator in a donor having died following brain death. Another possible aspect would consist in early characterization of renal grafts through analysis of mTOR and NRF2 expression. While access to this type of information would necessitate an invasive approach, one possible track would consist in analysis of renal cellular material in donor urine and blood; relevant assessments have been carried out in cancerology [26].

## 4. Materials and Methods

### 4.1. Experimental Model

All animal experiments were performed in accordance with the Care and Use of Laboratory Animals and Animal Research Reporting of In Vivo Experiments (ARRIVE) guidelines and approved by French Poitou-Charentes ethics committee of animal experimentation (protocol number CE2012-14, 15 November 2013). We used three-month-old Large White pigs weighing 40 ± 4 kg (MOPICT IBiSA Plateforme, INRA Magneraud, Surgères, France). 

### 4.2. Brain Death Model

12 pigs (large white, 35–52 kg) were utilized for the purposes of the study, which was conducted following the agreement of the Comité d’éthique de Poitou-Charentes (C2EA-84), protocol CE2012-14 in the surgical experimentation platform MOPICT (INRA, domaine du Magneraud, Surgères, France). Pre-medication was carried out using Midazolam IM 0.05 mg/kg 20 min prior to initiation of the experimentation. The pigs were anesthetized first by inhalation of a mixture of Sevoflurane 8% and oxygen. Following loss of consciousness, a 20 G catheter was set up on an ear vein and the anesthesia was deepened by injection of propofol (3 mg/kg), fentanyl (1 μg/kg), ketamine (1 mg/kg) and rocuronium (1 mg/kg). Respiratory tract management was ensured by installation of orotracheal intubation, size 6.5 (Hi-Contour, Mallinckrodt Medical^™^, Athlone, Ireland). The pigs were ventilated with a volume-controlled Servo 900C^™^ (Siemens, Solna, Sweden) with tidal volume set at 8 mL/kg, and the respiratory rate was adjusted to obtain PaCO2 between 35 and 40 mmHg and FiO2 at 50%. Positive end-expiratory pressure was 5 cm of H20, an I/E ratio set at 1/3. No alveolar recruitment maneuver was carried out. Maintenance was achieved through iterative administration of propofol at 3 mg/kg/h, fentanyl 1.5 μg/kg/h and rocuronium 1 mg/kg according to the animal’s behavior. A femoral arterial catheter and a central venous line in the internal jugular vein put into place surgically, and monitoring was then ensured by PICCO1^®^ Edward (Pulsion medical system, Irving, USA). A supra-pubic catheter was installed following vesical incision with hourly diuresis measurement. Transition to brain death (BD) was provoked by carving a hole at 10 cm from the coronal plane and 10 cm from the sagittal line at the level of the frontal zone, and afterwards by detachment of the dura mater and insertion of a Foley catheter Ch 18 in the epidural space; the catheter was then swollen with saline according to the Purins model. For the six animals in the SgBD (slowly generated brain death) group, brain death was provoked slowly by inflation of the Foley catheter balloon in accordance with a sequence of 5 mL at 0 min, followed by deflation of 5 mL at 10 min, inflation of 10 mL at 20 min, deflation of 5 mL at 30 min, inflation of 10 mL at 40 min, deflation of 5 mL at 50 min and inflation of 10 mL at 60 min up until brain death. For the six animals in the RgBD (rapidly generated brain death) group, brain death was rapidly provoked by inflation of the Foley catheter balloon of 20 mL in 5 min. The animals were monitored by a double-derivation electroencephalogram (EEG). Brain death diagnosis was carried out on the basis of clinical examination: A reactive bilateral mydriasis, absent corneal reflexes, abolition of spontaneous ventilation after hypercapnia response test and flat line EEG. Sedation was discontinued once brain death was validated. Reanimation consisted in maintaining mean arterial pressure above 70 mmHg, with normal hematosis under mechanical ventilation (tidal volume 8 mL/kg) and diuresis between 10 and 15 mL kg/h. Vascular filling with crystalloid solutions was guided by injection volume variation (IVV). Hemodynamic support was achieved by continuous intravenous infusion of noradrenaline. Occurrence of polyuria, which was attributed to central diabetes insipidus during brain death, was treated by low-dose desmopressin. A need to increase noradrenalin doses to a level exceeding 0.3 μg/kg/min occasioned administration of hydrocortisone at 1 mg/kg/h. Reanimation following brain death proceeded for 4 h.

Blood collections were performed at baseline time (before BD induction), at the time of BD declaration (BD time), and during donor management at 60 min after BD declaration (60 aBD), 120 min (120 aBD), 180 min (80 aBD) and 240 min (240 aBD). At 240 min after BD (4 h management) kidneys were collected. The left kidney was flushed with a cold University Wisconsin (UW) solution and preserved in cold UW in static condition during 18 h before transplantation. The right kidney was collected for biological analysis with biopsies at the end of donor management and at the end of kidney preservation in UW as previously described. The biological analyses of BD kidney were compared to control kidneys collected on three-month-old healthy pigs.

### 4.3. Kidney Allo-Transplantation and Renal Function Evaluation After Transplantation

**Figure 6:**
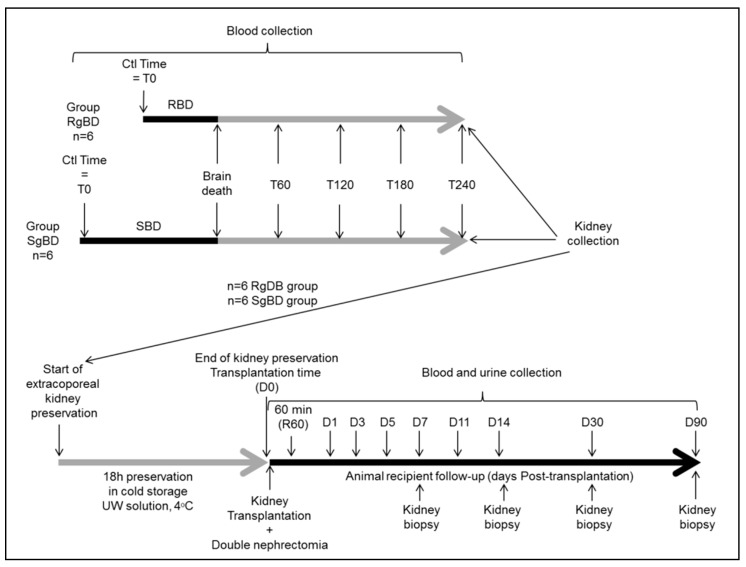
Experimental design.

At the end of static preservation, the left kidney was allotransplanted in a fully swine leukocyte antigen (SLA) compatible littermate, bilaterally nephrectomized pig (six with grafts of fast induction group and six with grafts of slow induction group). Heterotopic allotransplantation was performed in anaesthetized pig via midline incision. End-to-side aorta and inferior vena cava anastomoses were performed, just above the iliac bifurcation. Ureteroneocystostomy was carried out. After kidney transplantation, the pigs were placed in a metabolic cage for urine and blood collection during the first seven days and at days 11, 14 and 90 after transplantation (Figure 6). Blood collections were performed at day −1 (1 day before transplantation), 60 min after kidney reperfusion and at days 1 (D1), D3, D5, D7, D11, D14 and D90 after kidney transplantation. Follow-up was performed for 90 days, animals were anesthetized at D90 post-transplantation and the transplanted kidneys were collected for histological analysis.

### 4.4. Soluble Protein Quantification and Renal Function Evaluation

Blood NGAL, HMGB1 and IL18 proteins were assessed in porcine plasma using ELISA kit (respectively, Eurobio, Euromedex and Invitrogen France) according to the manufacturer’s instructions. Blood creatinine, sodium, lactate dehydrogenase (LDH) and aspartate aminotransferase (ASAT) were measured with a modular bioanalyzer (Roche-Diagnostics, France). Diuresis was evaluated by urine production after transplantation. Glomerular filtration rate (GFR) was calculated as: ((Diuresis mL/h)*urine creatinine)/blood creatinine = GFR L/h, and converted in GFR mL/min.

### 4.5. Histological Study

Biopsy samples from corticomedullary kidney collected at day 90 after transplantation were fixed with 4% formalin and paraffin-embedded. Histological interstitial fibrosis evaluated by red Sirius staining (collagen fibers I and III staining) was quantified with ImageJ software. Renal tubulitis and leukocyte infiltration were scored on histological HES staining.

### 4.6. Real-Time Quantitative PCR

Total RNAs from porcine cortical renal tissue were extracted with a commercial kit (Macherey-Nagel, Hœrdt, France). Genomic DNA was removed using DNA-free kit and first-strand reverse-transcription (Applied-Biosystems, Saint-Aubin, France) was performed. Real-Time qPCR assays were performed on a RotorGene Q (Qiagen, Courtaboeuf, France). Porcine DNA primers were designed using OligoPerfect™ (Life Technologies), QuantPrim (Max-Planck-Gesellschaft, Potsdam, Germany) and OligoAnalyser (Integrated DNA Technologies, Coralville, USA); the sequences are detailed in supplementary Table S1. RNA was expressed as “relative to expression in healthy ctl kidney” determined with the Pfaffl method (expressed as the relative fold change), using *β-actin, CYA62, SDHA, RPLPO* and *L19* genes as internal controls. 

### 4.7. Western Blot Analysis

Western Blotting was carried out using specific antibodies; Bcl-2 (D17C4, Cell Signaling, France), Bax (ab115193, Abcam, France), aconitase 2 (ab110321, Abcam, France), nitrotyrosine (05-233, Millipore, France), 4HNE (ab5605, Millipore, France), sirtuine-3 (D22A3, Cell signaling, France), sirtuine-1 (SC-9475, Cell signaling, France), phosphorylated sirtuine-1 (Sc-2314, Cell signaling, France), peroxisome proliferator-activated receptor gamma coactivator 1-alpha (PGC1a, ab54481, Abcam, France), nuclear factor (erythroid-derived 2)-like 2 (NRF2, ab31163, Abcam, France), mammalian target of rapamycin (mTOR, 2983S, Cell signaling, France), phospho mTOR (5536P, Cell signaling, France), eNOS total (610296, BD Transduction, Le Pont de Claix, France) and eNOS phophorylated Ser1177 (C9C3; Cell-Signaling, Saint Quentin-Yvelines, France). Protein quantification level was expressed as ratio of total proteins loaded on precast gels with stain-free technology (BioRad, France). All blots were obtained by imaging with chemidoc and quantificated with ImageLab software (BioRad).

### 4.8. Statistical Methods

Kinetic results are expressed in mean ± SD, and other results are expressed as a scatter plot with the median. Statistical analysis was performed with a Kruskal–Wallis test and Dunn’s post-test for kinetic parameters, and for other parameters statistical analysis was performed as Mann–Whitney test, with R and NCSS software, statistical significance was set at *p* < 0.05. *n* = 6.

## 5. Conclusions

Brain death kinetics influences early function recovery in graft and contributes to the intensity of fibrosis in a pig model of renal transplantation. The mechanism involved seems to be an allostatic response differing from response to the oxidative stress modulating the NRF2-mTOR system. Confirmation of these results might lead to proposal of new means of characterization and preconditioning of brain death donors.

## Figures and Tables

**Table 1:** Hemodynamic parameters evolution of SgBD and RgBD groups, before brain death (Baseline), at brain death (BD) and during 240 min of porcine management after brain death (aBD).

	Baseline	BD	60_aBD_	120_aBD_	180_aBD_	240_aBD_
**MAP_SgBD_. mmHg**	102 ± 4	160 ± 10 ^β^	78 ± 6	91 ± 14	75 ± 2	83 ± 3
**MAP_RgBD_. mmHg**	105 ± 2	146 ± 13 ^β^	68 ± 5	74 ± 3 ^£^	71 ± 4	72 ± 8
**CO_SgBD_. l/min**	6.7 ± 0.5	10.6 ± 3.3 ^β^	7. 0 ± 2.5	6.9 ± 2.2	7.7 ± 1.4	7.3 ± 2.3
**CO_RgBD_. l/min**	5.9 ± 1.4	11.3 ± 1.7 ^β^	7.7 ± 1.3	7.4 ± 1.2	6.5 ± 2.2	7.5 ± 2.0
**PaO_2 SgBD_. mmHg**	333 ± 97	334 ± 120	324 ± 75	254 ± 48	334 ± 103	301 ± 122
**PaO_2 RgBD_. mmHg**	365 ± 113	394 ± 134	374 ± 180	260 ± 63	405 ± 103	326 ± 127
**Hb_SgBD_. g/dL**	8.6 ± 0.5	11.6 ± 0.8	9.7 ± 1	10.2 ± 1.5	10.1 ± 1.5	10.5 ± 1.5
**Hb_RgBD_. g/dL**	9.0 ± 0.5	11.0 ± 1	10.1 ± 1	9.6 ± 1.6	10.3 ± 1.5	11 ± 1.5
**Lactate_SgBD_. mmol/L**	1.0 ± 0.4	2.4 ± 1.0 ^β^	3.4 ± 0.7 ^β^	2.5 ± 1.3	2.3 ± 1.8	2.2 ± 1.7
**Lactate_RgBD_. mmol/L**	0.9 ± 0.4	2.4 ± 1.0 ^β^	2.8 ± 1.2 ^β^	1.6 ± 0.6	1.3 ± 0.5	1.2 ± 0.3

MAP (mean arterial pressure), CO (cardiac output), PaO_2_ (blood oxygen partial pressure), Hb (hemoglobin), BD = brain death, aBD = after brain death. Results are expressed as mean ± SEM. Statistics: *p* < 0.05; ^β^ time vs. Baseline (T0) and ^£^ RgBD group vs. SgBD group.

## Supplementary Materials

Supplementary materials can be found at https://www.mdpi.com/1422-0067/20/15/3671/s1.

## Abbreviations

DBD	Brain Death Donor
EEG	Electroencephalogram
UW	University Wisconsin
LD	Linear dichroism
NRF2	Nuclear factor erythroid-2-related factor 2
mTOR	Mechanistic target of rapamycin
CCL2/MCP-1	Monocyte chemoattractant protein-1
eNOS	Endothelial nitric oxide synthase
SIRT-1	Sirtuin-1
PGC1-α	Peroxisome proliferator-activated receptor gamma coactivator 1-alpha
4-HNE	4-Hydroxynonenal
LDH	Lactate Deshydrogenase
ASAT	Aspartate aminotransferase activity
AUC	Area under the curve
