# Supplementary material for: Rapid or Slow Time to Brain Death? Impact on Kidney Graft Injuries in an Allotransplantation Porcine Model
Source: Int J Mol Sci. 2019 Jul 26;20(15):3671. doi: 10.3390/ijms20153671 (PMC6696377; doi:10.3390/ijms20153671)
Supplement: Supplementary file 1 [file ijms-20-03671-s001.pdf]

Table S1: Primers used for real-time RT-PCR in porcine renal cortex tissue

**Target**		**Primer Sequence**
L19 (RPL19)	Forward primer	5′- AATCGCCAACGCCAACTC -3′
	Reverse primer	5′- CAGCCCATCTTTGATCAGCTT -3′
RPLPO	Forward primer	5′- AGAAACTGTTGCCTCACATCC -3′
	Reverse primer	5′- CCTTATTGGCCAGCAGCA -3′
-Actin	Forward primer	5′- GATCGTGCGGGACATCAAG -3′
	Reverse primer	5′- GCCATCTCCTGCTCGAAGTC -3′
SDHA	Forward primer	5′- GAGTTCGTGCAGTTCCACCCTA -3′
	Reverse primer	5′- CCTCTCACCCTGGCTGTTGATA -3′
CYA62	Forward primer	5′- AAGACTGAGTGGTTGGATGG -3′
	Reverse primer	5′- AATGGTGATCTTCTTGCTGGT -3′
CCL-2	Forward primer	5′- TCTCCAGTCACCTGCTGCTAT -3′
	Reverse primer	5′- TGCTTCTTTAGGACACTTGCTG -3′
E-Selectin	Forward primer	5′- TCCAACCTTGCAGGATGATGTGC -3′
	Reverse primer	5′- TGCCAATACGCATTCAGCCCTTC -3′
ICAM-1	Forward primer	5′- GGCTGTGCACTGCAACAAGA -3′
	Reverse primer	5′- TGTGGCAATGCCAAATCCT -3′

Abbreviations: *L19* ribosomal protein gene (*RPL19*), ribosomal protein large *P0* (*RPLPO*), succinate dehydrogenase complex subunit *A* (*SDHA*), cyclophilin A *62* (*CyA62*), chemokine (C-C motif) ligand 2 (*CCL-2*), Endothelial Selectin (*E-Selectin*), Intercellular Adhesion Molecule 1 (*ICAM-1*)
